# Reference intervals for cd, hg, Mn and Pb in the general children population (3–14 years) of Kinshasa, Democratic Republic of Congo (DRC) between June 2019 and June 2020

**DOI:** 10.1186/s13690-023-01054-x

**Published:** 2023-03-15

**Authors:** Y. M. Tuakashikila, H. M. Mata, M. M. Kabamba, A. M. Malumba, J. K. Tuakuila

**Affiliations:** 1 Laboratory of Analytical Chemistry and Environmental Toxicology, Faculty of Sciences, University of Kinshasa, Kinshasa, Democratic Republic of Congo; 2 Faculty of Health Sciences, University of Sherbrooke, Quebec, Canada

**Keywords:** Blood metals, Urine metals, Reference intervals, Reference values, Children population, Kinshasa

## Abstract

The reference intervals (RIs), proposed by the International Federation of Clinical Chemistry and Laboratory Medicine (IFCC) and the International Union of Pure and Applied Chemistry (IUPAC), were derived for Cd, Hg, Mn and Pb in the blood and urine of the children population living in Kinshasa (*n* = 200, aged 3–14 years with 97 girls). Levels of metals were measured using coupled plasma mass (ICP-MS). In blood, the proposed RIs [P5-P95 (GM)] were 0.022–1.112 μg/L (0.074), 35.69–144.50 μg/L (71.43), 0.060 to 1.161 μg/L (0.208) and 6.597–15.740 μg/L (9.882) for Cd, Pb, Hg and Mn, respectively. Urinary levels [(P5-P95 (GM)] were 0.082–1.530 μg/L (0.366) for Cd, 1.827–18.500 μg/L (5.458) for Pb, 0.323–1.953 μg/L (0.709) for Hg and 0.070 to 1.703 μg/L (0.186) for Mn. As compared to the CDC updated blood Pb reference value (35 μg/L), Pb levels remain higher of public health concern. Cd and Mn levels were similar to those found in the same city in 2015 and databases involving non-occupationally exposed populations from other countries. Hg levels significantly lower than those found in the same city in 2015, probably due to exclusion criteria of metal exposure applying in the present survey (occupationally exposed to the studied metals, smoking habits, amalgam tooth fillings, fish consumption habit more than one time per week, etc.). These background metal exposures will be useful for future occupational and/or environmental surveys as well as undertaking a reliable regulation of chemical exposure in Kinshasa via a national HBM program.

## Introduction

Cadmium (Cd), manganese (Mn), mercury (Hg) and lead (Pb) are ubiquitous toxic metals due to their presence in soil, dust, uptake food and drinking water [1–8]. Cd is toxic carcinogenic to humans and responsible for kidney damage and bone demineralisation and fractures [2, 3, 9]. Pb is a neurological, haematological, renal, and gastrointestinal toxic element [5, 8, 10–12]. Furthermore, it is toxic to reproduction and probably carcinogenic to humans [13, 14]. Hg is mainly toxic to central nervous system and reproduction [15, 16]. Mn is an essential element required for humans [17, 18]. However, under certain high-dose exposure conditions, it can cause severe neurologic impairment, including a debilitating neurodegenerative disease [17–19].

As compared to adults, children are more vulnerable to toxic metals because of their hand-mouth behavior, diet, metabolic and physiologic characteristics [20–22]. Furthermore, children in disadvantaged households are at increased risk of exposure to many environmental hazards (e.g., toxic metals) exhibiting stronger health impairments even at lower levels of exposure compared to adults [23–26].

To provide useful quantitative information regarding the actual exposure of a population to these toxic metals as well as data regarding the resulting health effects and/or population susceptibility to chemical pollutants, Human Biomonitoring (HBM) is an important tool used to provide aggregated data of the exposures by targeting the chemicals of concern and their metabolites in biological matrices, such as blood and urine [23, 27–30]. HBM data can be used to establish reference ranges for selected chemicals in the general population or in specific targeted populations [29, 31–33]. This HBM approaches is excellent way of elucidating the distribution of chemical exposures and locating exposure dangerous zones as well as exposures among different age, sex and zone groups [34, 35]. According to the recommendations of the International Federation of Clinical Chemistry and Laboratory Medicine (IFCC) and the International Union of Pure and Applied Chemistry (IUPAC), reference intervals (RIs) indicate background exposure to chemical substances in a reference population [36–38].

In the Democratic Republic of Congo (DRC), the use of artisanal, illegal as well as legal mining activities leads to an increasing exposure of population to many metals of health concern in the mining zones of several settings [39–42]. There is a need to establish and update reference values as well as background levels for metals that are routinely measured for HBM to assess environmental and occupational exposures. Although Kinshasa, the capital and largest city of the DRC, is not considered as a mining zone, worrying exposure to some trace elements of health concern was reported in this population, previously [21, 42]. The present study is motivated by these findings and it aims at providing the Cd, Hg, Mn and Pb levels in 200 children, in order to establish RIs for the general children population living in Kinshasa. A conclusion will be given by providing recommendations to create a reliable regulation of chemical exposure as suggested in several countries.

## Methods

### Study population and data collection

Two stages have been used to construct a systematic sampling approach as published elsewhere [21, 43, 44]. Of the 22 administrative entities of urban area Kinshasa, 11 were selected randomly from the list. Parents of eligible children (220 such as 20 by administrative entity) received a detailed explanation of study procedures before consenting to participate [healthy boy and girl subjects between 3 and 14 years, living in Kinshasa ≥12 months, without amalgam tooth fillings, fish consumption habit >one time per week, smoking tobacco environment, etc.]. Positive responses were obtained from 90% (200 subjects) of those approached. Using the same methods of recruitment, 50 additional children living in the 2 rural areas of Kinshasa were also included (25 by administrative entity). Enrollment was implemented between June 2019 and June 2020. The research protocol was approved by the Bio-ethics Committee of the School of Public Health at the University of Kinshasa.

### Data collection

Two hundred children were invited to come to the local health center to provide both venous blood in 10 mL metal free tubes containing K_2_EDTA (aged 3–14 years) and urine in 20 mL metal-free polypropylene containers (aged > 5 years), and stored at −20C, as described elsewhere [34, 45]. Information collected in the questionnaires were socio-demographics, smoking habit, and lifestyle. These children were not hospitalized and were free of disease as assessed by interview and clinical examination. The samples were stored at -20 °C at the Analytical chemistry and Environmental toxicology laboratory of the University of Kinshasa. All samples were then kept frozen and transported in a cool box under dry ice to be analyzed at the Institut national de santé publique du Québec (INSPQ) using validated analytical methods [37, 46]. Normalizing a urinary biomarker concentration to urinary creatinine is often used to minimize the influence of the urinary flow rate in 24 h (urine dilution) on the urinary level of the biomarker [31, 44]. Urinary creatinine was determined on a Beckman Synchron LX 20 analyser (Beckman Coulter GmbH, Krefeld, Germany) by the Jaffe method [47].

### Analytical methods

Metals were measured in both blood and urine samples using coupled plasma mass (ICP-MS) methods [46, 48, 49]. For quality control purposes, rigorous quality control protocols and tested standard or certified reference materials were carried out. Briefly, blood samples were diluted in a basic solution (octylphenol ethoxylate and ammonium hydroxide) and urine samples were diluted in an acid solution (0.5% nitric acid) according to the methods performed by INSPQ [50]. Internal quality control was ensured by analyzing 2 or 3 different reference materials from the Québec Multielement External Quality Assessment Scheme (QMEQAS) in each analysis sequence. Furthermore, INSPQ has participated in proficiency testing for metals analyses in biological specimens (Table 1).

**Table 1:** Analytical methods

Biomarker		Method	CV	LOD	% < LOD	QA/QC
Cd	Blood	ICP-MS	< 10%	0.09 μg/L	28.5	G-EQUAS. LAMP, PCI, QMEQAS
	Urine			0.04 μg/L	33.2	
Hg	Blood	ICP-MS	< 15%	0.20 μg/L	30.9	
	Urine			0.04 μg/L	22.3	
Mn	Blood	ICP-MS	< 15%	0.043 μg/L	2.5	
	Urine			0.032 μg/L	34.8	
Pb	Blood	ICP-MS	< 10%	0.17 μg/dL	0.9	
	Urine			0.029 μg/L	6.6	

RIQAS (www.randox.com); G-EQUAS (www.g-equas.de); *QC* quality control, *QA* quality assurance scheme. www.cetac.com. LAMP- the U.S. Centers for Disease Control and Prevention’s Lead and Multielement Proficiency Program; *PCI* Interlaboratory Comparison Program for Metals in Biological Matrices, *QMEQAS* Quebec Multielement External Quality Assessment Scheme, *CV* inter-assay coefficient of variation, *LOD* limit of detection

### Statistical analysis

Statistical data analysis was completed using Prism GraphPad 9.41 (GraphPad Soft - ware, San Diego, CA, USA). The normality of residuals was evaluated using Kolmogorov–Smirnov test for continuous variables. For the descriptive statistics, results are presented as percentage for categorical variables and arithmetic means (± standard deviation), geometric means (95% confident intervals), percentiles (P25, P50, P75, P95) and minimum-maximum for continuous variables. Differences between groups were analyzed with analysis of variance (ANOVA), t-test, and trend test after log transformation of skewed variables. Differences in proportions were analyzed with chi-square test. Stepwise multiple linear regression analyses of log-transformed data were used to estimate factors influencing the investigated parameters (probability F to enter < 0.05 and probability F to remove > 0.10). All results were assessed for data quality based on the coefficient of variation (CV). The level below the limit of detection (LOD) was assigned a value of LOD/2 for statistical calculations [51, 52]. For biomarkers measured in urine, individuals with urine creatinine values < 0.3 g/L or > 3.0 g/L were removed prior to testing the other exclusion variables [53].

## Results and discussion

Considering that their biological systems and organs at various stages of development and less advanced elimination of contaminants, children are particularly sensitive to adverse effects caused by exposure [20, 23]. Characteristics of participants are presented in Table 2 with N, age, sex and percentages or arithmetic means with SD for subgroups.

**Table 2:** Demographic characteristics of the participants (3–14 years, Kinshasa between June 2019 and June 2020)

Subjects		Zone		P
		Urban	Rural	
		200	50	
Age (years)	Min-Max (AM±SD)	3–14 (8 ± 3)	3–14 (7 ± 2)	0.28
	n (% of 3–5)	40 (20)	15 (30)	0.03
Sex	n (% of girls)	97 (48.5)	26 (52.0)	0.10

*p p*-value (two-sided *p* < 0.05 was considered statistically significant), *AM* Arithmetic means, *SD* Standard deviation, *Min* minimum and *Max* maximum

Age of these children (*N* = 200) was between 3 and 14 years (20% were aged between 3 and 5 years old) and 8 years on average (SD: 3). 48.5% of the participants were girls.

For comparison, 50 additional children from rural zone were included. Although the higher proportion of group 3–5 observed in rural zone (30% vs 20%, *p* = 0.03), restricting the age group can provide a more homogeneous group with a more similar health status or reproductive age group [34, 53]. Descriptive results of the metal analyses blood and urine for the general children population of Kinshasa are provided in Tables 3 and 4, respectively. The GM, 5th percentile and 95th percentile level estimates had CVs under 15%. Consequently, the RIs derived from this 5th percentile (lower limit) and 95th percentile (upper limit) estimates should be considered as relevant [29, 33, 53]. The RIs produced can help to expand HBM studies in DRC and to plan research about the relationship between environmental exposures and human health effects. The affecting variables as well as the metals levels in blood and urine samples are discussed in the following sections.

**Table 3:** Blood Cd, Hg, Mn and Pb levels (μg/L) of the participants (3–14 years, Kinshasa between June 2019 and June 2020)

Elements	P5	P25	P50	P75	P95	AM±SD	GM (CI 95%)
Cd	0.022	0.067	0.072	0.101	0.112	0.083 ± 0.034	0.074 (0.069–0.080)
Hg	0.060	0.061	0.200	0.541	1.161	0.347 ± 0.355	0.208 (0.177–0.245)
Mn	6.597	8.242	9.890	11.540	15.740	10.240 ± 2.834	9.882 (9.486–10.290)
Pb	3.569	5.699	6.998	9.389	14.450	7.750 ± 3.300	7.143 (6.714–7.599)

*AM* Arithmetic means, *GM* Geometric means, *SD* Standard deviation, *CI95* 95% Confident intervals, Percentiles (P5, P25, P50, P75, P95)

**Table 4:** Urinary Cd, Hg, Mn and Pb levels (μg/L or μg/g of creatinine) of the participants (3–14 years, Kinshasa between June 2019 and June 2020)

Elements		P5	P25	P50	P75	P95	AM±SD	GM (CI 95%)
Cd	μg/L	0.082	0.210	0.365	0.625	1.453	0.483 ± 0.382	0.366 (0.296–0.452)
	μg/g of creatinine	0.142	0.221	0.322	0.445	0.761	0.359 ± 0.185	0.317 (0.277–0.363)
Hg	μg/L	0.323	0.509	0.640	0.925	1.953	0.913 ± 1.143	0.709 (0.602–0.835)
	μg/g of creatinine	0.243	0.413	0.577	0.995	1.577	0.787 ± 0.868	0.614 (0.519–0.727)
Mn	μg/L	0.070	0.070	0.170	0.307	1.703	0.510 ± 1.643	0.186 (0.140–0.247)
	μg/g of creatinine	0.023	0.073	0.138	0.244	1.244	0.948 ± 5.254	0.161 (0.115–0.225)
Pb	μg/L	1.676	3.130	5.775	9.433	17.680	7.028 ± 5.328	5.458 (4.486–6.640)
	μg/g of creatinine	1.827	2.893	4.453	7.208	18.500	5.976 ± 5.422	4.731 (3.987–5.613)

*AM* Arithmetic means, *GM* Geometric means, *SD* Standard deviation, *CI95* 95% Confident intervals, Percentiles (P5, P25, P50, P75, P95)

Sex, age, and zone background are common internal exposure determinants for describing exposure to metals. Globally, except blood Pb levels which decreased with increasing age, GM Cd, Hg, Pb levels were increased with age as reported by several other authors for adults [37, 54, 55]. No sex difference was observed in Cd, Hg and Mn levels as reported elsewhere [55–57]. However, GM Pb blood levels were higher in boys than in girls which is consistent with findings of previous studies and reflecting higher exposure levels [5, 7, 49, 55, 56, 58, 59]. Not significant difference between urban and rural populations was found for all metals as reported elsewhere [21, 40, 55].

### Reference intervals (RIs) for cd, hg, Mn and Pb levels

#### Cd levels

For HBM of Cd exposure, primarily the determination of Cd in urine, but also of Cd in blood is applied [60, 61]. Cd in blood reflects both recent and cumulative exposures [3, 9, 62]. The proposed RIs (P5-P95 (GM), 0.022–1.112 μg/L (0.074) (Table 5) which were similar to those reported in the same city previously [21] and consistent with findings of other surveys such as German [(P25-P95: 0.06–0.23 μg/L [49] and 0.40–1.07 μg/L) [49, 55], Canadian [(P95 (GM): 0.20 μg/L (0.099)) [46], US [(P50-P95: 0.100–0.220 μg/L) [62] and Correa [(P95: 0.90 μg/L) [64] databases (Table 5). Cd in urine reflects life-time exposure [7]. The background measured in Kinshasa [(P5-P95 (GM), 0.082–1.530 μg/L (0.366)] were similar to 0.18–1.7 μg/L (0.34) found in the same city [21] but higher than databases presented in Germany [(P25-P95 (GM): 0.08–0.24 μg/L (0.072, 51], Czech Republic [(P50(GM):0.096 μg/L (0.096) [63], Canada [(P95 (GM): 0.28 μg/L (0.018) [46] and USA [(P50-P95: 0.036–0.148 μg/L [62].

**Table 5:** Cd, Hg, Mn and Pb levels (μg/L) in different international settings

Reference	Settings (Ages)	Matrix	Percentile	Cd	Hg	Mn	Pb
This study	Kinshasa (Ages 3–14)	Blood	P5-P95 (GM)	0.022–1.112 (0.074)	0.060–1.161 (0.208)	6.597–15.740 (9.882)	35.69–144.50 (71.43)
		Urine		0.082–1.53 (0.366)	0.323–1.953 (0.709)	0.070–1.703 (0.186)	1.827–18.500 (5.458)
Tuakuila et al. [21]	Kinshasa (Ages 3–14)	Blood	P25-P95 (GM)	0.09–4.2 (0.14)	1.3–4.2 (1.7)	–	45–146 (59)
		Urine		0.18–1.7 (0.34)	5.5–19.3 (6.5)	–	2.9–23.0 (5.7)
Vogel et al. [48]	Germany (Ages 3–13)	Blood	P50-P95 (GM)	0.06–0.23 (−)	–	–	9.4–19.9 (9.47)
		Urine		0.08–0.24 (0.072)	0.07–0.25 (0.067)	–	–
Burn et al. [55]	Germany (Ages 3–11)	Blood	P50-P95	0.40–1.07	1.80–3.68	–	12.3–21.4
Health Canada [46]	Canada (Ages 3–11)	Blood	P10-P95 (GM)	0.097–0.20 (0.099)	0.34–1.9 (0.34)	–	2.6–12.0 (5.0)
		Urine		0.047–0.28 (0.048)	–	–	–
CDC [62]	USA (Ages 3–11)	Blood	P50-P95	0.100–0.220	0.262–1.710	10.5–17.9	6.20–20.02
		Urine		0.036–0.148	0.241–0.570	0.133–0.410	0.460–1.19
SZÚ [63]	Czech Republic (Ages 3–11)	Urine	P50 (GM)	0.096 (0.096)	0.28 (0.276)	–	–
Jeong et al. [64]	Republic of Korea (Ages 3–13)	Blood	P50	0.90	–	–	14.3

Percentiles (P5, P25, P50, P75, P95), GM: Geometric means

#### Pb levels

Pb in blood is a highly reliable biological marker of recent exposure to Pb [5, 54, 65]. The proposed RIs [P5-P95(GM)] were 35.69–144.50 μg/L (71.43). These results were with line of those found in 2015 by Tuakuila et al. [21] but higher than those reported elsewhere in Table 5. In 2021, CDC updated the blood Pb reference value to 3.5 μg/dL which provides an opportunity for additional progress in addressing longstanding disparities in lead exposure and BLLs in children [66]. Consequently, the background Pb exposure measured in this study constitute a major public health concern [5, 40, 65–68]. Although excretion of absorbed Pb occurs primarily in urine, urine Pb is less preferred as a maker of exposure to Pb [5, 10]. Children of Kinshasa provided Pb background [(P5-P95 (GM)] ranging from 1.827 to 18.500 μg/L (5.458). These data agreed with a previous survey in Kinshasa which shown similar Pb levels (P25-P95 (GM): 2.9–23.0 μg/L (5.7) [21]. However, these results were higher as compared to databases involving US children: P50-P95: 0.460–1.19 μg/L [62]. Pb-contaminated air, Pb-based paint, Pb-contaminated soil, Pb-contaminated food and Pb-containing dust constitute the most common sources of Pb exposure in the general Kinshasa population [21, 40, 55, 68].

#### Hg levels

Hg in Blood primarily reflect recent exposures [15, 16]. Hg results [P5-P95(GM)] were 0.060 to 1.161 μg/L (0.208). In line with these results, several databases were reported in literature [Germany (P50-P95: 1.80–3.68 μg/L [55], Canada [(P50-P95: 0.34–1.9 μg/L (0.34) [46] and USA [(P50-P95: 0.262–1.710 μg/L [62]. Regarding these, the background Hg exposures in Kinshasa were lower than those reported in Kinshasa by Tuakuila et al. [21] (Table 5). In urine, Hg represents primarily exposure to elemental Hg and its inorganic [69]. The results proposed as RIs[P5-P95(GM)]: 0.323–1.953 μg/L (0.709)] were in line with the results from other countries [Germany (P25-P95 (GM): 0.07–0.25 μg/L (0.067, 51], Czech Republic [(P50(GM):0.28 μg/L (0.276) [63] and USA (P50-P95: 0.241–0.570 μg/L [62] but significantly lower than those found in 2015 by Tuakuila et al. [21] [5.5–19.3 μg/L (6.5)], It is probably due to some exclusion criteria of Hg exposure applying in the present study (amalgam tooth fillings, fish consumption habit).

#### Mn levels

Mn in whole-blood is useful in investigating Mn nutritional status as well as monitoring Mn exposure in the general population [17, 18, 70]. Estimation results for the background Mn exposure were 6.597–15.740 μg/L (9.882). The finding largely agrees with a previous background analysis of NHANES in USA [(P50-P95: 10.5–17.9 μg/L] [62]. Mn in urine represents the excretion of excess Mn from the body and elevated levels may indicate occupational exposure or excessive nutritional intake [17, 18]. In this study, the Mn RIs [(P5-P95 (GM)] were 0.070 to 1.703 μg/L (0.186). These findings could be reflected in the results from other countries, such as USA (P50-P95: 0.133–0.410 μg/L (0.123) [62].

A major limitation should be considered in evaluating present results. With regard to study population and data collection. The sample collection methods used here were not robust but by chance, which were practically inevitable under present survey conditions and susceptible to errors associated with sample collection. Moreover, potential contamination by environmental tobacco smoking, alcohol habits and sampling conditions was not assessed.

## Conclusion

This study provides the reference intervals for Cd, Hg, Mn and Pb in the blood and urine of the children population living Kinshasa. Pb levels measured remain higher of public health concern as compared to the CDC updated blood Pb reference value (35 μg/L). However, current background levels of Cd, Hg and Mn show similar levels as compared to databases involving non-occupationally exposed populations from other countries. The findings of this study suggest to undertake a reliable regulation of chemical exposure in Kinshasa via a national HBM program.

## Data Availability

Not applicable. However, the study results will report to individuals sample donors with proper explanations.
